# Tocilizumab and Cytokine Release Syndrome in COVID-19 Pneumonia: Experience From a Single Center in Pakistan

**DOI:** 10.7759/cureus.20219

**Published:** 2021-12-06

**Authors:** Muhammad Hassan, Fibhaa Syed, Maria Zafar, Mansoor Iqbal, Naveed Ullah Khan, Hafiza Faiza Mushtaq, Mazhar Badshah

**Affiliations:** 1 Neurology, Shaheed Zulfiqar Ali Bhutto Medical University, Islamabad, PAK; 2 Internal Medicine, Shaheed Zulfiqar Ali Bhutto Medical University, Islamabad, PAK; 3 Obstetrics and Gynaecology, Shaheed Zulfiqar Ali Bhutto Medical University, Islamabad, PAK; 4 Neurology, Pakistan Institute of Medical Sciences, Islamabad, PAK

**Keywords:** cytokine release syndrome, interleukin-6 (il-6), covid-19, sars-cov-2, tocilizumab

## Abstract

Background

Tocilizumab (TCZ), an interleukin-6 (IL-6) receptor blocker, emerged as a treatment for cytokine release syndrome (CRS) in patients with severe COVID-19 pneumonia. The main objective of the study is to discuss the treatment response of TCZ in severe and critically ill patients with COVID-19 pneumonia. Patient demographics, laboratory parameters before and after TCZ therapy, and clinical outcomes in 20 patients in a single center were prospectively reviewed.

Results

Out of 120 patients, 96 (80%) were males and 24 (20%) were females. Only eight (10%) patients did not have any previously known comorbidity. There were 78 (65%) patients with severe disease, while 42 (35%) have critically severe disease. Of the 120 patients, only 36 required a second dose of TCZ in our study based on clinical background. Neutrophils and C-reactive protein (CRP) levels were observed to be raised in all patients, while lymphopenia was observed in 114/120, and D-dimer levels were elevated in 102 (85%) patients. After the second dose of tocilizumab, 102 (85%) patients reduced oxygen requirement within four days, and 14 patients were removed on the second dose of tocilizumab on clinical grounds. Of these 120 patients, in two weeks, 30 (25%) were discharged. Within three weeks, 60 of them were discharged, while 12 were discharged after three weeks, and 18 patients died in our study despite treatment.

Conclusion

TCZ appeared to be a good treatment option in patients with CRS and severe and critical pneumonia, and for patients with raised IL-6 levels despite single TCZ therapy, a repeat dose is recommended.

## Introduction

The pandemic caused by SARS-CoV-2 infection has transformed the world into a hybrid era of information, treatment, and way of living since December 2019. Patients who died of this fatal infection were found to have extremely high pro-inflammatory markers (interleukin-6 (IL-6)) in their first clinical case reports. In this initial clinical-pathological process of SARS-CoV-2 infection, bilateral diffuse alveolar exudative injury with CD4 and CD8 cell reduction and an upsurge of Th17 cells on the injury site are observed [1]. IL-6 and IL-23 play a pivotal role in the stimulation of Th17 cells and the maturation of Th0 cells [2].

SARS-CoV-2 pneumonia-related cytokine release syndrome (CRS) is attributed to the significant release of IL-6, tumor necrosis factor-α (TNF-α), and IL-12 [3,4]. CRS usually advanced quickly in patients and lead them to cardiac collapse, multiple organ dysfunction, and death. There is an immense need for early detection and treatment of CRS. Tocilizumab (TCZ), an interleukin-6 (IL-6) receptor humanized monoclonal antibody, is therefore recommended by the Novel Coronavirus Diagnosis and Treatment of Pneumonia guidelines by the National Health Commission of China for severely ill patients with elevated IL-6.

TCZ can be used for extensive bilateral opacities or in critical patients with elevated IL-6 levels with first dose of 4-8 mg/kg (diluted in 100 mL normal saline with a maximum dose of 800 mg/dose) and can be repeated after 12 hours based on the initial response [5]. It was hypothesized that TCZ may reduce mortality and shift to high oxygen demand in SARS-CoV-2 ARDS. In this prospective observational study, we aimed to perceive the clinical picture and response of patients to TCZ regime therapy.

## Materials and methods

Study design, setting, and participants

This observatory study recruited all patients with COVID-19 infection who received TCZ treatment at the Shaheed Zulfiqar Ali Bhutto Medical University in Islamabad from April 28 to June 22, 2020. The study was approved by the Shaheed Zulfiqar Ali Bhutto's Ethical Committee of Islamabad Medical University (F.1-1/2015/ERB/SZABMU/554).

Data collection

Data on preformed performa with demographics, comorbidities, diagnosis, laboratory results, and clinical outcomes were collected. All patients were divided into asymptomatic or presymptomatic infection, mild illness, moderate illness, severe illness, and critical illness based on the National Institute of Health guidelines (in our hospital, we only admit those who required oxygen therapy, and the remaining are all sent to top quarantine center). Before and after administration of TCZ, C-reactive protein (CRP), D-dimer, serum ferritin, and IL-6 were obtained. IL-6, CRP, D-dimer, and serum ferritin (acute-phase reactants) were defined as elevated when they were higher than 7 pg/mL, >5 mg/dL, >250 mg/dL, and >300 ng/mL in males and >150 ng/mL in females, respectively. Study dropouts were defined as those patients whose laboratory or clinical data were missing before and after TCZ administration. CRP or IL-6 values up to 48 hours before TCZ administration were selected as the value before TCZ therapy, and the changes of the value after TCZ administration were observed for a week. Clinical outcomes were observed one week after TCZ administration. Involvement of different systems will be noted along with pulmonary systems, such as CNS and cardiac involvement.

Statistical analysis

SPSS version 23.0 was used to carry out statistical analysis. Data were displayed as numbers and percentages, if applicable. A p-value of less than 0.05 was considered statistically relevant.

## Results

Baseline characteristics

The mean age was 58.7 ± 13.5 years, and most of the patients were above 60 years (Table 1). Of the 120 patients, 96 (80%) were male and 24 (20%) were females, and 78 (65%) patients were evaluated as severe and 42 (35%) were critical. Among the comorbidities of all patients, the most prevalent were diabetes and hypertension, with 42 (35%) and 30 (25%) patients, respectively, while 24 (20%) patients had both diabetes and hypertension. Apart from pulmonary manifestation in 90 (75%) patients, 18 (15%) patients had CNS involvement and 12 (10%) patients had multiorgan involvement. Among radiological findings, 42 (35%) patients had ground-glass opacities bilaterally, 54 (45%) patients had a ground-glass appearance with areas of consolidation, and 24 (20%) patients had a ground-glass appearance with septal thickening. All patients were treated with oxygen therapy, including dual oxygen therapy in 60 (50%) patients, face mask in 30 (25%) patients, non-rebreather mask in 24 (20%) patients, and noninvasive ventilation in six (5%) patients (Table 1).

**Table 1:** Demographic characteristics of the patients on presentation µ = Range of values of the patients £ = Non-rebreather mask € = Face mask + non-rebreather mask

Characteristics	Patients (n = 120)
Age	
20–40 years	6 (5%)
41–60 years	54 (45%)
>60 years	60 (50%)
Gender	
Male	96 (80%)
Female	24 (20%)
Comorbidities	
Diabetes mellitus (DM)	42 (35%)
Hypertension (HTN)	30 (25%)
Malignancy	6 (5%)
HTN and DM	24 (20%)
HTN and ischemic heart disease	6 (5%)
No comorbidity	12 (10%)
Main system of involvement	
Pulmonary system	90 (75%)
Central nervous system	18 (15%)
Multiorgan involvement	12 (10%)
State of illness	
Severe	78 (65%)
Critical	42 (35%)
HRCT findings	
Ground-glass opacities bilaterally	42 (35%)
Ground-glass opacities with areas of consolidation	54 (45%)
Ground-glass opacities with septal thickening	24 (20%)
Laboratory test before tocilizumab	
Total leukocyte count (TLC)	11854 ± 3297.05 (6670–18500)^µ^
Neutrophils (percentage %)	87.83 ± 4.43 (78–94.70)^µ^
Lymphocytes (percentage %)	10.6 ± 4.37 (4.5–21.30)^µ^
Alanine aminotransferase (ALT) (IU/L)	41.4 ± 13.26 (18–64)^µ^
Creatinine (mg/dL)	1.11 ± 0.58 (0.40–2.4)^µ^
Creatine phosphokinase (CPK) (IU/L)	265.05 ± 262.53 (29–920)^µ^
Ferritin (ng/mL)	1606.6 ± 1093.03 (289–5504)^µ^
D-Dimer (ng/mL)	808.05 ± 585.02 (50–2192)^µ^
C-Reactive protein (CRP) (mg/L)	110.54 ± 114.6 (11.60–510)^µ^
Interleukin-6 (IL-6)	161.6 ± 299.5 (17–1420)^µ^
Oxygen therapy	
Face mask	30 (25%)
NRM^£^	24 (20%)
Dual supply^€^	69 (50%)
CPAP/BiPAP	6 (5%)
Adverse effects after tocilizumab	0
Clinical outcome	
Discharge from the hospital	102 (85%)
Death	18 (15%)
Hospitalization days	
≤14 days	30 (25%)
14–21 days	60 (50%)
>21 days	12 (10%)

Laboratory examination

Table 2 shows the results of baseline laboratory tests before and after TCZ. Thirty (25%) patients have an abnormal WBC count (mean: 11854 ± 3297.05/L). Neutrophilia was observed in all patients (mean: 87.83% ± 4.43%), while lymphopenia was observed in 114/120 patients (mean: 10.6% ± 4.37%). The D-dimer level was elevated in 85% of patients (102/120) (mean: 808.05 ± 585.02 ng/mL). CRP levels increased in all patients (mean: 110.54 ± 114.6 mg/L).

Sixty patients had an elevated CPK (mean: 265.05 ± 262.53 IU/L). Of the 120 patients, 36 had slightly elevated ALT (mean: 41.4 ± 13.26 IU/L). All patients were also screened for IL-6 (mean: 161.6 ± 299.5 pg/mL) and serum ferritin (mean: 1606 ± 1093.03 ng/mL) before and after TCZ.

**Table 2:** Laboratory tests before and after tocilizumab β = Not all patients received a second dose of tocilizumab. Out of 120 patients, only 36 patients were eligible candidates for the second dose of tocilizumab on clinical grounds. µ, ¥, and ē = range of laboratory parameters after and before the first and second dose of TCZ, respectively.

Laboratory parameters	Before tocilizumab	First dose of tocilizumab (n = 20)	Oxygen demand after first dose of tocilizumab	Second dose of tocilizumab (n = 6)^β^	Oxygen demand after second dose of tocilizumab
Total leukocyte count (TLC)/L	11854 ± 3297.05 (6670–18500)^µ^	9507.5 ± 3977.62 (1630–16500)^¥^	Nasal cannula = 6 (5%)	9590 ± 2528.4 (6480–13500)^ē^	Face mask = 24 (66.67%)
Neutrophils (percentage %)	87.83 ± 4.43 (78–94.70)^µ^	83.75 ± 6.3 (69–92)^¥^	Face mask = 36 (30%)	81.8 ± 4.8 (75–89)^ē^	Non-rebreather mask = 12 (16.67%)
Lymphocytes (percentage %)	10.6 ± 4.37 (4.5–21.30)^µ^	14.64 ± 6.32 (7–30)^¥^	Non-rebreather mask = 48 (40%)	17.15 ± 4.7 (10.20–23.9)^ē^	Dual oxygen = 6 (16.67%)
Alanine aminotransferase (ALT) (IU/L)	41.4 ± 13.26 (18–64)^µ^	54.4 ± 15.71 (38–102)^¥^	Dual oxygen supply (nasal cannula and non-rebreather mask) = 18 (15%)	68.8 ± 17.8 (55–100)^ē^	CPAP = 0
Creatinine (mg/dL)	1.11 ± 0.58 (0.40–2.4)^µ^	1.06 ± 0.47 (0.50–2.20)^¥^	CPAP = 6 (5%)	1.3 ± 0.63 (0.80–2.50)^ē^	
Creatine phosphokinase (CPK) (IU/L)	265.05 ± 262.53 (29–920)^µ^	227 ± 234.05 (30–790)^¥^		123.5 ± 132.1 (28–360)^ē^	
Ferritin (ng/mL)	1606.6 ± 1093.03 (289–5504)^µ^	1533.85 ± 997.6 (299–4390)^¥^		1349.2 ± 363.98 (960–1865)^ē^	
D-Dimer (ng/mL)	808.05 ± 585.02 (50–2192)^µ^	709.4 ± 557.58 (25–1990)^¥^		611 ± 394.6 (10–1186)^ē^	
C-Reactive protein (CRP) (mg/L)	110.54 ± 114.6 (11.60–510)^µ^	62.07 ± 54.59 (7.80–249)^¥^		39.32 ± 55.99 (2.5–145)^ē^	
Interleukin-6 (IL-6)	161.6 ± 299.5 (17–1420)^µ^	181.2 ± 344.43 (10–1296=5)^¥^		225.33 ± 375.53 (49–990) ^ē^	

Imaging features

All patients presented with abnormal HRCT findings as ground-glass appearance, plague, and abnormal foci in peripheral zone with septal thickening and subpleural area (Table 1), and no pleural and mediastinal lymphadenopathy or pulmonary emboli have been reported in all patients.

Treatment outcomes

During the intervening days, clinical symptoms were simultaneously significantly mitigated. The peripheral saturation of oxygen significantly improved. After the second dose of tocilizumab, 102 (85%) patients reduced oxygen requirement within four days. Of the 48 patients, no additional oxygen therapy was needed. A major improvement in lymphocyte numbers, ALT, creatinine, D-dimer, serum ferritin, CRP, and IL-6 levels was observed during the treatment of tocilizumab after the first dose. The second dose of tocilizumab was not given to 84 patients on clinical grounds as shown in Table 2. Of these, in two weeks, 30 (25%) patients were discharged. Within three weeks, 60 of them were discharged after tocilizumab (Table 1).

Safety

All adverse events reported by patients have been registered. No significant events of tocilizumab were observed in our research. Several records have been made of some adverse drug reactions, such as elevated transaminase, neutropenia, and inflammation. During the treatment, there was no emergent bacterial, fungal, or viral infection.

## Discussion

We studied the effects of tocilizumab (IL-6 inhibitor) in 120 critically ill patients who were brought in suffering from COVID-19 pneumonia. The results emphasized that immediate treatment with this drug thereafter resulted in drastic improvement in the signs and symptoms of the disease, such as hypoxemia and multiple opacities on the chest radiograph. This proposes that tocilizumab can be an effective treatment.

COVID-19 is an emerging global pandemic producing milder symptoms during the early course of the disease. In the majority of the population, the symptoms adhere to a more serious phase of the disease manifesting as tightness of the chest and severe shortness of breath and, even in many cases, leading to respiratory compromise and failure. One of the findings of such patients is the presence of multiple opacities on the chest imaging. Such patients always require ambulatory oxygen therapy and in many cases an intubation-ventilation in the critical care unit. Sadly, 4.3%-11% died despite the recommended treatment in the literature [6]. Moreover, the mortality rate reached as high as 60.5% of the population [7]. In our study, all the 120 patients have had the standard routine treatment for at least a week before tocilizumab was opted but were affected with severe hypoxemia, fever, and worsening of chest opacities. Once the IL-6 inhibitor was given, body temperatures and the respiratory system showed marked improvement in most of the patients. The feeling of chest tightness was relieved, followed by normal oxygen saturations and a lesser need for oxygen intake. Twelve of our patients were extubated and weaned off the ventilator within five days. This suggests that treating with IL-6 inhibitor in all those patients having persistent high-grade fever, multiple diffuse lung opacities, and higher titers of IL-6 levels would result in a quicker relief of symptoms and control the progression of the disease to its life-taking form [8].

A downward trend in the lymphocytes has been studied as an important aspect of diagnoses and also gives clues about the severity of COVID-19 [8]. In our study, we saw a lesser number of lymphocytes in about 85% of the patients (102/120) and in some returned to normal in about 52.6% of the patients (60/114) after treating with tocilizumab for five days. Another finding was the decrease of CRP levels to normal. The levels of IL-6 were high in all the patients before initiating treatment. Once treated, the levels will remain high for a few days as their receptors have been sabotaged by tocilizumab. Similar events are observed in chimeric antigen T-cell treatment [9]. In all 120 patients who were given TCZ first dose, there is a markedly downward trend in CRP, D-dimer, ferritin, and TLC, especially in CRP (110.54 to 62.07 mg/L after the first dose and then to 39.32 mg/L after the second dose of TCZ) and ferritin (1606.6 to 1533.85 ng/mL after the first dose of TCZ and then to 1349.2 ng/mL after the second dose of TCZ) as evident in Table 2.

In 114 (90.5%) of our patients, the lung opacities had gone. This suggests that the lung parenchyma needs enough time for repair. During the treatment phase, no severe adverse reactions and pulmonary infections were observed. The signs and symptoms of the patients improved to a great extent with a better prognosis. All the patients were discharged within two weeks of treatment with tocilizumab. Hence, this drug can treat severe forms of this disease, as explained by the fact that IL-6 blockade halts the febrile and inflammatory cytokine storm phase of the disease. Our study has several limitations and shortcomings, such as the limited number of patients and a single observation study, which could possibly lead to arguments and biased opinions. The strength of this evidence should be enhanced. To acquire more evidence, a randomized control trial should be designed. Many of the studies are under consideration explaining the mechanism of IL-6 in COVID-19.

## Conclusions

To summarize our findings, tocilizumab may improve clinical outcomes and suppresses the severity and deterioration of the disease especially in terms of oxygen therapy in severely ill patients. This emphasizes new revolutionary ideas, treatment modalities, and a therapeutic strategy related to cytokine release syndrome and prompts testing in a randomized controlled trial.

## Appendices

Abbreviations

IL-6: interleukin-6

CD4: cluster of differentiation 4

Th17: helper T-cell 17

TNF: tumor necrosis factor

CRP: C-reactive protein

TCZ: tocilizumab

Registration trial

This study was a registered clinical trial with ID NCT04873141 on clinicaltrail.gov.

## References

[1] Xu Z, Shi L, Wang Y (2020). Pathological findings of COVID-19 associated with acute respiratory distress syndrome. Lancet Respir Med.

[2] Miossec P, Kolls JK (2012). Targeting IL-17 and TH17 cells in chronic inflammation. Nat Rev Drug Discov.

[3] Li Y, Chen M, Cao H, Zhu Y, Zheng J, Zhou H (2013). Extraordinary GU-rich single-strand RNA identified from SARS coronavirus contributes an excessive innate immune response. Microbes Infect.

[4] Lau SK, Lau CC, Chan KH (2013). Delayed induction of proinflammatory cytokines and suppression of innate antiviral response by the novel Middle East respiratory syndrome coronavirus: implications for pathogenesis and treatment. J Gen Virol.

[5] Colaneri M, Bogliolo L, Valsecchi P (2020). Tocilizumab for treatment of severe COVID-19 patients: preliminary results from SMAtteo COvid19 REgistry (SMACORE). Microorganisms.

[6] Chen N, Zhou M, Dong X (2020). Epidemiological and clinical characteristics of 99 cases of 2019 novel coronavirus pneumonia in Wuhan, China: a descriptive study. Lancet.

[7] Yang X, Yu Y, Xu J (2020). Clinical course and outcomes of critically ill patients with SARS-CoV-2 pneumonia in Wuhan, China: a single-centered, retrospective, observational study. Lancet Respir Med.

[8] Wang D, Hu B, Hu C (2020). Clinical characteristics of 138 hospitalized patients with 2019 novel coronavirus-infected pneumonia in Wuhan, China. JAMA.

[9] Tanyi JL, Stashwick C, Plesa G, Morgan MA, Porter D, Maus MV, June CH (2017). Possible compartmental cytokine release syndrome in a patient with recurrent ovarian cancer after treatment with mesothelin-targeted CAR-T cells. J Immunother.

